# Supplementary material for: Prediction of late blight severity in a large panel of potato genotypes using low-altitude aerial images and machine learning methods
Source: PLoS One. 2026 Nov 3;21(11):e0347446. doi: 10.1371/journal.pone.0347446 (PMC13730388; doi:10.1371/journal.pone.0347446)
Supplement: Supplementary file 2 [file pone.0347446.s006.docx]

**Response to comments made by reviewers to the manuscript PONE-D-26-16165R1 entitled: Prediction of late blight severity in a large panel of potato genotypes using low-altitude aerial images and machine learning methods**

We would like to thank the editor for the comments provided. We agree with the recommendations and have revised the manuscript accordingly. Below, we address the Journal requirements and the Additional Editor comments, in the same order in which they were presented:

**REPLIES TO JOURNAL REQUIREMENTS:**

1. If the reviewer comments include a recommendation to cite specific previously published works, please review and evaluate these publications to determine whether they are relevant and should be cited. There is no requirement to cite these works unless the editor has indicated otherwise.

Our response:

We confirm that we have not received any recommendation from the reviewers to cite specific previously published works.

2. Please review your reference list to ensure that it is complete and correct. If you have cited papers that have been retracted, please include the rationale for doing so in the manuscript text, or remove these references and replace them with relevant current references. Any changes to the reference list should be mentioned in the rebuttal letter that accompanies your revised manuscript. If you need to cite a retracted article, indicate the article’s retracted status in the References list and also include a citation and full reference for the retraction notice.

Our response:

We have carefully reviewed our reference list to ensure it is complete and correct. We confirm that none of the cited references have been retracted. During this review, we identified and corrected the following issues:

1) **Duplicate reference removed:** The following reference had been inadvertently duplicated in the previously revised version (R1) of the reference list:

**Reference 39/41 (duplicate):** “Gitelson AA, Kaufman YJ, Merzlyak MN. Use of a green channel in remote sensing of global vegetation from EOS-MODIS. Remote Sens Environ. 1996;58: 289–298. doi:10.1016/S0034-4257(96)00072-7”

This duplication has been corrected, and the reference now appears only once in the reference list (as reference 40), and is properly cited in the text. As a result, the total number of references decreased from 78 to 77.

2) **Uncited reference now correctly cited in the text:** The following reference was present in the reference list (previously as reference 38, now reference 39) but was not cited anywhere in the manuscript text:

Barnes EM, Clarke TR, Richards SE, Colaizzi PD, Haberland J, Kostrzewski M, et al. Coincident detection of crop water stress, nitrogen status and canopy density using ground-based multispectral data. Proceedings of the 5th International Conference on Precision Agriculture and Other Resource Management, July 16-19, 2000, Bloomington, MN, USA. 2000 [cited 19 Nov 2022]. Available: <http://hdl.handle.net/10113/4190>

This reference has now been cited in the Materials and Methods section, as detailed below.

3) **Citation order corrected**: We identified that some in-text citations in the submitted manuscript were not properly numbered according to their true order of first appearance. This inconsistency has now been corrected throughout the text. The text has been revised as follows:

Page 10, line 236:

"First, the plot-level mean values of four widely used vegetation indices—the Normalized Difference Vegetation Index (NDVI; [37]), Normalized Difference Red Edge (NDRE; [38]), Red Edge reflected band (RE, [39]), and Green Normalized Difference Vegetation Index (GNDVI; [40])—were computed for each LB severity observation dataset."

4) **Updated dataset references:** Two dataset references were updated by the data repository after the submission of the previously revised version (R1). Their DOIs and position in the reference list remain unchanged, but the following updates were made:

**Reference 31:** The dataset title was updated from "Replication data for: Late blight severity in potato breeding clones from CIP's LB-resistant and heat-tolerant breeding population" to "Replication data for: late blight severity in breeding clones from CIP's LBHTC2 breeding population."

**Reference 32:** The author order was corrected from "Lindqvist-Kreuze H, Aponte M" to "Aponte M, Lindqvist-Kreuze H."

The updated references are as follows:

31. Gastelo M, Quispe K, Bastos M, Pozo J, Erquinio E, Vega R, et al. Replication data for: late blight severity in breeding clones from CIP's LBHTC2 breeding population. International Potato Center; 2026. doi:10.21223/P3/PN2RGR
32. Aponte M, Lindqvist-Kreuze H. Replication data for: late blight severity in potato accessions from the CIP genebank core collection. International Potato Center; 2026. doi:10.21223/P3/HJLUJZ

**REPLIES TO ADDITIONAL EDITOR COMMENTS:**

The two potato trials (Trial A and Trial B) were conducted in Oxapampa, Pasco, Peru, but why there are so great differences between the environments including temperature, humidity and precipitation in these two trials? This point should be described in the text.

Our response:

Thank you for this comment. There are no substantial differences in the environmental conditions between the two trials. Both were conducted at the same location, planted in parallel plots but offset by approximately one month. Temperature and humidity were similar between trials, only precipitation differed significantly, due to higher rainfall in January and February. This higher rainfall affected Trial B only, since Trial A had already been harvested by that time, as it was planted earlier. However, we recognize that the original wording in the manuscript could lead to misunderstanding, so it has been reworded as follows:

Page 5, line 111:

“During the crop growing season in both trials, meteorological conditions were characterized by minimum temperatures of 8.6–16.2 °C, maximum temperatures of 18.9–27.0 °C, and relative humidity of 69.0–94.7% (S2 Fig). Due to the 1-month offset between trials, total precipitation was higher in Trial B (894.7 mm) than in Trial A (559.6 mm), driven by higher precipitation in January–February (S2 Fig).”

Additionally, in the description of Supplementary Figure 2, we identified an error in the date range of the climatic conditions. The text has been rewritten as follows:

Page 29, line 764:

“**S2 Fig. Weather conditions during potato trials and UAV acquisitions**. Weather conditions in the study area of Oxapampa, Peru (September 2019–February 2020). Minimum (blue lines) and maximum (red lines) temperatures are shown on the left y-axis, while precipitation (PP, cyan bars) and relative humidity (RH, black dotted lines) are shown on the right y-axis. Vertical green and orange dashed lines mark planting and harvest dates for the first (A) and second (B) trials, respectively. Red arrows indicate the two unmanned aerial vehicle (UAV) flight dates for multispectral imagery acquisition.”

It is better to delete Figure 1. This Figure is not necessary, because it provides limited information.

Our response:

Thank you for this suggestion. We have moved Figure 1 to the Supporting Information as a supplementary figure, since it still provides useful context for readers. The numbering of the subsequent figures has been corrected accordingly.

In the manuscript, ‘Both trials were conducted according to standard agronomic practices [15] until all plants had emerged and grown sufficiently large for disease assessment, which was performed 40 days after planting (DAP).’, and then ‘LB severity assessments were conducted weekly at the plot level, comprising seven assessments in Trial A (starting at 41 DAP) and five assessments in Trial B (starting at 38 DAP).’, what is the relationship between ‘which was performed 40 days after planting (DAP)’ and ‘(starting at 41 DAP)’ or ‘(starting at 38 DAP)’, especially the relationship between ‘which was performed 40 days after planting (DAP)’ and ‘(starting at 38 DAP)’?

Our response:

We agree with the reviewer's comment and thank them for pointing out this lack of clarity. As indicated in the international cooperators' guide [15], LB severity readings should begin as soon as weather conditions become conducive to disease development, and should start immediately once symptoms are observed. The onset of LB severity assessments in both trials was determined by the appearance of initial LB symptoms on the susceptible control varieties (spreader rows), which occurred at 40 DAP in Trial A and 37 DAP in Trial B. Weekly LB severity evaluations began the following day in each trial (41 DAP in Trial A and 38 DAP in Trial B). We acknowledge that the previous wording did not clearly convey this relationship, and the text has been revised accordingly, as follows:

Page 7, line 153:

"Both trials were conducted according to standard agronomic practices until all plants had emerged and grown sufficiently large for disease assessment [15]. To prevent early LB infection and before allowing natural LB infestation, fungicide Dithane M-45 WP NT (Mancozeb, BASF®, Peru) was applied preventively twice, at 80% and at full emergence."

Page 8, line 175:

“LB severity assessments were conducted weekly at the plot level, comprising seven assessments in Trial A and five assessments in Trial B. The onset of disease assessment was defined by the appearance of initial LB symptoms on the susceptible control variety (Yungay), which occurred at 40 DAP in Trial A and 37 DAP in Trial B. Weekly evaluations began the following day in each trial (41 DAP in Trial A and 38 DAP in Trial B). The weekly interval was selected according to the standardized CIP methodology for late blight field trials and the expected rate of epidemic development under the relatively cool conditions at the Oxapampa site [15].

Now there are still some problems in the References section, e.g., the format of the titles of the journals and the format of the first letter of each word in each reference title. Each reference in the References section should be checked carefully according to the requirements of the Journal. Please review your reference list to ensure that it is complete and correct.

Our response:

Thank you for this observation. We have carefully reviewed the entire reference list and corrected the following formatting issues, in accordance with PLOS ONE's requirements:

- **Journal name abbreviations:** All journal titles have been standardized using the official National Library of Medicine (NLM) journal title abbreviations.
- **Title capitalization:** All reference titles have been corrected to sentence case, capitalizing only the first word and proper nouns, consistent with Vancouver style requirements.
- **Other corrections:** We also identified and fixed additional minor errors.
