# Supplementary material for: Prediction of late blight severity in a large panel of potato genotypes using low-altitude aerial images and machine learning methods
Source: PLoS One. 2026 Nov 3;21(11):e0347446. doi: 10.1371/journal.pone.0347446 (PMC13730388; doi:10.1371/journal.pone.0347446)
Supplement: Supplementary file 1 [file pone.0347446.s005.docx]

**Response to comments made by reviewers to the manuscript PONE-D-26-16165 entitled: Prediction of late blight severity in a large panel of potato genotypes using low-altitude aerial images and machine learning methods**

We would like to thank you for the thorough and very constructive review, which we found agree with the recommendations and have changed the corresponding parts of the manuscript accordingly. All the editor and reviewer’s comments were addressed and responded in the same order, as follows:

**REPLIES TO JOURNAL REQUIREMENTS:**

1. Please ensure that your manuscript meets PLOS ONE's style requirements, including those for file naming. The PLOS ONE style templates can be found at

https://journals.plos.org/plosone/s/file?id=wjVg/PLOSOne_formatting_sample_main_body.pdf and

https://journals.plos.org/plosone/s/file?id=ba62/PLOSOne_formatting_sample_title_authors_affiliations.pdf

Our response:

We thank the editor for this comment. We have revised the manuscript and all associated files to comply with PLOS ONE's style requirements, including file naming conventions, as specified in the provided templates.

**2.** We note that Figure 1 in your submission contain map images which may be copyrighted. All PLOS content is published under the Creative Commons Attribution License (CC BY 4.0), which means that the manuscript, images, and Supporting Information files will be freely available online, and any third party is permitted to access, download, copy, distribute, and use these materials in any way, even commercially, with proper attribution. For these reasons, we cannot publish previously copyrighted maps or satellite images created using proprietary data, such as Google software (Google Maps, Street View, and Earth). For more information, see our copyright guidelines: <http://journals.plos.org/plosone/s/licenses-and-copyright>.

We require you to either (1) present written permission from the copyright holder to publish these figures specifically under the CC BY 4.0 license, or (2) remove the figures from your submission:

1. You may seek permission from the original copyright holder of Figure 1 to publish the content specifically under the CC BY 4.0 license.

We recommend that you contact the original copyright holder with the Content Permission Form (http://journals.plos.org/plosone/s/file?id=7c09/content-permission-form.pdf) and the following text:

“I request permission for the open-access journal PLOS ONE to publish XXX under the Creative Commons Attribution License (CCAL) CC BY 4.0 (http://creativecommons.org/licenses/by/4.0/). Please be aware that this license allows unrestricted use and distribution, even commercially, by third parties. Please reply and provide explicit written permission to publish XXX under a CC BY license and complete the attached form.”

Please upload the completed Content Permission Form or other proof of granted permissions as an "Other" file with your submission.

In the figure caption of the copyrighted figure, please include the following text: “Reprinted from [ref] under a CC BY license, with permission from [name of publisher], original copyright [original copyright year].”

2. If you are unable to obtain permission from the original copyright holder to publish these figures under the CC BY 4.0 license or if the copyright holder’s requirements are incompatible with the CC BY 4.0 license, please either i) remove the figure or ii) supply a replacement figure that complies with the CC BY 4.0 license. Please check copyright information on all replacement figures and update the figure caption with source information. If applicable, please specify in the figure caption text when a figure is similar but not identical to the original image and is therefore for illustrative purposes only.

The following resources for replacing copyrighted map figures may be helpful:

USGS National Map Viewer (public domain): http://viewer.nationalmap.gov/viewer/

The Gateway to Astronaut Photography of Earth (public domain): http://eol.jsc.nasa.gov/sseop/clickmap/

Maps at the CIA (public domain): https://www.cia.gov/library/publications/the-world-factbook/index.html and https://www.cia.gov/library/publications/cia-maps-publications/index.html

NASA Earth Observatory (public domain): http://earthobservatory.nasa.gov/

Landsat: http://landsat.visibleearth.nasa.gov/

USGS EROS (Earth Resources Observatory and Science (EROS) Center) (public domain): http://eros.usgs.gov/#

Natural Earth (public domain): http://www.naturalearthdata.com/

Our response:

We thank the editor for this comment. We have replaced the Google Satellite basemap previously used in Figure 1 with the following CC BY 4.0-compatible sources:

(1) for the orthomosaic panels (Trial A and Trial B), we now use a Sentinel-2 Level-2A satellite image (product S2B_MSIL2A_20210214T151659_N0500_R125_T18LVP_20230518T174713), acquired on 14 February 2021 and freely available through the Copernicus Data Space Ecosystem (ESA); and

(2) for the inset map of Peru, we replaced the Google Satellite background and the administrative boundaries (department and province) with public-domain data from Natural Earth (<https://www.naturalearthdata.com/>). The figure caption has been updated to specify all data sources.

The figure 1 caption has been updated to specify all data sources.

**Page 7, Materials and Methods section, Line 151:**

Fig 1. The study area. Location of the two potato trials in Oxapampa, Pasco, Peru: Trial A, comprising 2,745 breeding clones with two replications over 0.9 ha, and Trial B, comprising 492 genebank accessions with three replications over 0.3 ha. Basemap imagery for Trial A and Trial B: Sentinel-2 L2A (ESA/Copernicus, acquired February 14, 2021). Basemap and administrative boundaries (Peru; Pasco department; Oxapampa province) in the inset map: Natural Earth (public domain, <https://www.naturalearthdata.com/>).

**3.** In your Methods section, please provide additional information regarding the permits you obtained for the work. Please ensure you have included the full name of the authority that approved the field site access and, if no permits were required, a brief statement explaining why

Our response:

Thank you for your suggestion. No governmental or special collection permits were required for this study, as the work did not involve protected or endangered plant species, access to protected natural areas, or the introduction, movement, or artificial inoculation of the pathogen; potato materials were instead evaluated under naturally occurring LB infection. Field trials were conducted on farmer’s fields in Oxapampa, Pasco, Peru, rented by the International Potato Center (CIP) under a land-use agreement with the local landowner, a common practice for CIP's late blight (LB) resistance screening program, as Oxapampa is a locality with historically high natural LB pressure (Gastelo et al., 2022; Forbes et al., 2014; Pérez et al., 2001 — references already cited in the manuscript). We have added this information to the Materials and Methods section, as detailed below.

**Page 6, Materials and Methods section, Line 129:**

“Field trials were conducted on farmer’s field rented by the International Potato Center (CIP) under a land-use agreement with the local landowner, as Oxapampa has been traditionally used by CIP as a screening site for LB resistance [5, 15, 26].”

**Page 7, Materials and Methods section, Line 138:**

“These environmental conditions allowed evaluation of the potato materials under naturally occurring LB infection, without artificial inoculation.

**Page 7, Materials and Methods section, Line 145:**

“No governmental or special collection permits were required for this study.”

**4.** Thank you for stating the following financial disclosure:

"This research was supported, in whole or in part, by the Bill & Melinda Gates Foundation (grant number OPP1070785); the CGIAR Research Program on Roots, Tubers, and Bananas (RTB); the One CGIAR Initiative on Plant Health; and the CGIAR Sustainable Farming Science Program and the Breeding for Tomorrow Science Program. CGIAR is a global research partnership dedicated to transforming food, land, and water systems in the face of the climate crisis and advancing a food-secure future."

Please state what role the funders took in the study. If the funders had no role, please state: "The funders had no role in study design, data collection and analysis, decision to publish, or preparation of the manuscript."

If this statement is not correct you must amend it as needed.

Please include this amended Role of Funder statement in your cover letter; we will change the online submission form on your behalf.

Our response:

We thank the editor for this comment. Upon review, the CGIAR Research Program on Roots, Tubers, and Bananas (RTB) and the One CGIAR Initiative on Plant Health were removed from the financial disclosure, and additional funding sources specific to each trial were added. The revised financial disclosure reads as follows:

“This research was supported, in whole or in part, by the Bill & Melinda Gates Foundation (grant number OPP1070785); the CGIAR Sustainable Farming Science Program; the Breeding for Tomorrow Science Program; the United States Agency for International Development (USAID) through Window 3 support to the International Potato Center (CIP) under the CGIAR Trust Fund (MTO No. 069033), Crops to End Hunger (CtEH): Potato project (Amendment No. 9 to the Contribution Agreement, 2020); and the European Union’s Horizon 2020 research and innovation programme, under the project G2P-SOL "Linking genetic resources, genomes and phenotypes of Solanaceous crops" (grant agreement No. 677379). CGIAR is a global research partnership dedicated to transforming food, land, and water systems in the face of the climate crisis and advancing a food-secure future.”

Role of Funder statement:

- The CGIAR Sustainable Farming Science Program and the Breeding for Tomorrow Science Program provided support by funding part of the staff time involved in the development of the manuscript.
- The Bill and Melinda Gates Foundation (BMGF; grant number OPP1070785) provided support through equipment acquired during the implementation of the project, which was subsequently used in this study, including GPS devices, a PAR sensor, and a datalogger.
- Trial A was funded by USAID through Window 3 support to CIP under the CGIAR Trust Fund (MTO No. 069033), CtEH: Potato project (Amendment No. 9 to the Contribution Agreement, 2020).
- Trial B was conducted under the G2P-SOL project, with financial support from the European Union Horizon 2020 research and innovation programme (grant agreement No. 677379).

The funders had no role in the design of the study; in the collection, analysis, or interpretation of data; in the writing of the manuscript; or in the decision to publish the results.

**5**. If the reviewer comments include a recommendation to cite specific previously published works, please review and evaluate these publications to determine whether they are relevant and should be cited. There is no requirement to cite these works unless the editor has indicated otherwise.

Our response:

Thank you for your comment. The reviewer did not make this recommendation.

**6**. Please review your reference list to ensure that it is complete and correct. If you have cited papers that have been retracted, please include the rationale for doing so in the manuscript text, or remove these references and replace them with relevant current references. Any changes to the reference list should be mentioned in the rebuttal letter that accompanies your revised manuscript. If you need to cite a retracted article, indicate the article’s retracted status in the References list and also include a citation and full reference for the retraction notice.

Our response:

We thank the reviewer for this comment. We have carefully reviewed our complete reference list against retraction databases (e.g., Retraction Watch) and publisher records and confirm that none of the cited articles have been retracted. No changes to the reference list were required as a result of this check.

During this review, we also identified a minor inconsistency in reference formatting: references #53, #57, and #70 in the original submission (Kamoun & Smart, 2005; Van Damme et al., 2012; and Araus & Cairns, 2014, respectively — now renumbered as #55, #59, and #72 in the revised reference list following the addition of new citations) included PubMed PMID/PMCID identifiers, while the remaining references did not. For consistency across the reference list, we have removed these identifiers so that all references now follow a uniform format. No content, authors, or bibliographic data were altered — only the optional PMID/PMCID fields were removed for formatting consistency.

In addition, in response to a subsequent reviewer request, two new references were added to the manuscript:

- Garry G, Forbes GA, Salas A, Santa Cruz M, Pérez WG, Nelson RJ. Genetic diversity and host differentiation among isolates of *Phytophthora infestans* from cultivated potato and wild solanaceous hosts in Peru. Plant Pathology. 2005;54:740–8. doi:10.1111/j.1365-3059.2005.01250.x
- Lindqvist-Kreuze H, Gamboa S, Izarra M, Pérez W, Correa MY, Astete A, et al. Population structure and host range of the potato late blight pathogen *Phytophthora infestans* in Peru spanning two decades. Plant Pathology. 2020;69:334–46. doi:10.1111/ppa.13125

Finally, the two CIP Dataverse datasets cited in the manuscript have been updated, and their corresponding citations have been revised accordingly:

Previously:

- Gastelo M, Quispe K, Bastos M, Pozo J, Erquinio E, Vega R. Dataset for: Breeding for resistance to late blight - LBHTC2 population- Phenotypic and Genomic Selection [Internet]. International Potato Center; 2020 [cited 2024 Feb 21]. Available from: <https://data.cipotato.org/dataset.xhtml?persistentId=doi:10.21223/GCJRH6> doi:10.21223/GCJRH6
- Lindqvist-Kreuze H, Aponte M. Dataset for: Genome wide association study (GWAS) to identify favourable alleles for late blight resistance in core collection [Internet]. International Potato Center; 2021 [cited 2024 Feb 27]. Available from: <https://data.cipotato.org/dataset.xhtml?persistentId=doi:10.21223/YT4UZU> doi:10.21223/YT4UZU

Now:

- Gastelo M, Quispe K, Bastos M, Pozo J, Erquinio E, Vega R, et al. Replication Data for: Late blight severity in potato breeding clones from CIP's LB-resistant and heat-tolerant breeding population. International Potato Center; 2026. doi:10.21223/P3/PN2RGR
- Lindqvist-Kreuze H, Aponte M. Replication Data for: Late blight severity in potato accessions from the CIP genebank core collection. International Potato Center; 2026. doi:10.21223/P3/HJLUJZ

These updates have been reflected accordingly in the reference list and corresponding in-text citations of the revised manuscript.

Additional Editor Comments:

The data in the manuscript should be checked carefully. In line 337, ‘Trials A (1,476 plots) and B (5,490 plots)’ is correct?

‘vegetation indices’ in the Keywords should be replaced with ‘vegetation index’’.

Each reference in the References section should be checked carefully according to the requirements of the Journal.

Our response:

**Comment 1:** *In line 337, 'Trials A (1,476 plots) and B (5,490 plots)' is correct?*

We thank the editor for this comment. The description in Table 1 contains a labeling error: Trial A and Trial B were inadvertently swapped. Trial A consisted of 2,745 potato clones with 2 replications, resulting in 5,490 plots; Trial B consisted of 492 potato accessions with 3 replications, resulting in 1,476 plots (as described in the "Plant material and experimental design" subsection of Materials and Methods). We have corrected the Table 1 caption accordingly to read "Trials A (5,490 plots) and B (1,476 plots)".

**Page 17, Results section, line 380:**

“Table 1. Pearson correlation (R) values between mean vegetation indexes—Normalized Difference Vegetation Index (NDVI), Green Normalized Difference Vegetation Index (GNDVI), Red Edge reflected (RE), and Normalized Difference Red Edge (NDRE)—and Late Blight severity observations from Trials A (5,490 plots) and B (1,476 plots).”

In addition, thanks to this comment, we identified a related error in the "Late blight estimation" subsection, where Trial A was incorrectly described as having 5,790 grids; this has been corrected to 5,490 grids, consistent with one grid per plot.

**Page 9, Materials and Methods section, line 231:**

“Subsequently, polygonal grids were created, labeled, and assigned to each plot according to the experimental design, resulting in 5,490 grids in Trial A and 1,476 grids in Trial B per dataset.”

**Comment 2:** *'vegetation indices' in the Keywords should be replaced with 'vegetation index'.*

We thank the editor for this comment. We will update the Keywords field in the submission system accordingly, replacing "vegetation indices" with "vegetation index" upon submission of the revised version.

**Comment 3:** *Each reference in the References section should be checked carefully according to the requirements of the Journal.*

We thank the editor for this comment. We have carefully reviewed the References section and revised it to comply with the Journal's citation style. As detailed in our response to requirement 6 of the editor's initial comments, this review included verifying that no cited references have been retracted, correcting a minor formatting inconsistency (removal of PMID/PMCID identifiers for consistency across the list), incorporating two new references requested by a reviewer, and updating two CIP Dataverse dataset citations to reflect their finalized records.

**REPLIES TO REVIEWER’S COMMENTS**

**Reviewer #1:**

The abstract is well-structured, but it could be slightly shortened to improve clarity and readability, especially in the methodology section.

Our response:

We thank the reviewer for this positive assessment and for the helpful suggestion. We have revised the abstract to make it more concise while preserving all key elements of the study. The abstract has been shortened from 300 to 244 words. No results, methodological details, or quantitative information were removed; the revisions are limited to the elimination of redundancy and the simplification of sentence structure. We believe the abstract now reads more fluidly and that the methodological section is clearer and easier to follow. All changes are marked in the revised manuscript.

The introduction provides strong background; however, some sentences are lengthy and may benefit from simplification for better flow.

Our response:

We are grateful to the reviewer for recognizing the strength of the background provided and for the constructive suggestion regarding readability. We have carefully revised the Introduction to improve its flow, focusing on the sentences that carried the heaviest syntactic load. No references, concepts, or supporting arguments were removed; all citations remain unchanged and the logical structure of the Introduction is preserved. The revised Introduction is 81 words shorter and, in our view, reads more fluidly. All changes are marked in the revised manuscript.

The objectives are clearly stated, but they could be presented more concisely to enhance focus.

In the methodology, additional clarification on image preprocessing steps would improve reproducibility.

Our response:

**Comment 1:** *The objectives are clearly stated, but they could be presented more concisely to enhance focus*.

We thank the reviewer for this observation. The statement of objectives at the end of the Introduction has been rewritten in a more concise form. Both objectives remain unchanged in substance and scope; the revision affects only their formulation, which is now shorter and, in our view, gives the reader a sharper sense of the focus of the study. In revising this section, we also took the opportunity to clarify the distinction between the two experimental units (an early-generation breeding trial and a genebank core collection), which had previously been referred to jointly as "breeding trials." This clarification was applied consistently throughout the manuscript.

**Page 5, Introduction section, line 113, before:**

“Specifically, the study’s objectives are as follows: i) to quantify the extent to which aerial phenotyping captures genetic variation in LB resistance, by comparing image-derived indicators with conventional field assessments across diverse genotypes; ii) to analyze the potential reduction of temporal assessments when UAV-based imagery is used to monitor genotypes' LB severity.”

**Page 5, Introduction section, line 113, now:**

“Specifically, we aimed to: i) quantify how well aerial phenotyping captures genetic variation in LB resistance, by comparing image-derived indicators with conventional field assessments; ii) determine whether UAV imagery can reduce the number of temporal assessments needed to monitor LB severity.”

**Comment 2:** *In the methodology, additional clarification on image preprocessing steps would improve reproducibility*

We thank the reviewer for this suggestion. We have expanded the "Field late blight severity assessment, image acquisition, and preprocessing" subsection to provide additional detail on the radiometric and geometric processing steps performed in Pix4Dmapper, and clarified the connection to the subsequent plot-level reflectance extraction, which is described in the following "Late blight severity estimation" subsection. In addition, as noted in our response to Reviewer 2's comment on irradiance variation during image acquisition, we incorporated real-time monitoring of irradiance stability using a PAR sensor and datalogger, selecting the most stable image set for each assessment date. These clarifications are detailed below.

Text added to manuscript:

**Page 10, Materials and Methods section, line 213:**

“Given the frequent occurrence of passing clouds at the study site, irradiance stability during image acquisition was monitored in real time using a LI-190S quantum (PAR) sensor connected to a LI-1400 datalogger (LI-COR, Lincoln, USA). For each assessment date, multiple aerial image sets were acquired, and the set exhibiting the most stable irradiance conditions was selected for radiometric correction and subsequent analysis.”

**Page 10, Materials and Methods section, line 218:**

… “version 4.8.4;”

**Page 10, Materials and Methods section, line 219:**

“Radiometric calibration combined the pre- and post-flight reference panel images with continuous irradiance measurements from the DLS/PAR sensor within the Pix4Dmapper radiometric correction workflow. Vignetting and lens distortion were corrected automatically within Pix4Dmapper, …”

**Page 10, Materials and Methods section, line 223:**

“Plot- and pixel-level reflectance values were subsequently extracted from these orthomosaics, as described in the following subsection.”

Some figures (e.g., workflow diagrams) could be made more self-explanatory with clearer labels and captions.

Our response:

We thank the reviewer for this comment. We have revised Figure 2 (workflow diagram) by adding more descriptive labels to each processing step and expanding the figure caption accordingly. Additionally, we improved the visual presentation of Figure 4 and Supplementary Figure S2 by adjusting marker size and transparency in the scatter plots to better convey data density in regions of high point overlap. Furthermore, we took this opportunity to submit all figures to PLOS's Figure Analysis tool to verify compliance with the journal's figure requirements.

Minor grammatical and typographical errors are present and should be carefully checked throughout the manuscript.

Our response:

We thank the reviewer for this comment. We have carefully proofread the entire manuscript and corrected the grammatical and typographical errors identified.

The discussion is comprehensive, but reducing repetition between results and discussion sections would improve readability.

Our response:

We thank the reviewer for this suggestion. We have revised the Discussion section to reduce repetition of specific results already reported in the Results section, focusing instead on interpretation. The following changes were made:

**Page 21, Discussion section, line 462, before:**

"Consistent with this principle, NDVI showed strong associations with LB severity in this study. The identification of threshold values (0.6 and 0.55) enabled objective classification of healthy and diseased canopy tissues across assessment periods. Similar threshold-based approaches have proven effective for disease severity detection in several crops [63–66], supporting the robustness of vegetation index-derived metrics for plant disease monitoring."

**Page 21, Discussion section, line 462, now**:

"Consistent with this principle, the identification of trial-specific NDVI thresholds enabled objective classification of healthy and diseased canopy tissue across assessment periods, in line with similar threshold-based approaches reported for disease severity detection in other crops [63–66], supporting the robustness of vegetation index-derived metrics for plant disease monitoring."

**Page 22, Discussion section, line 472, before:**

"The KRR-based model for estimating LB severity (LB-KRR) further captured nonlinear relationships within multispectral reflectance data, leading to consistently improved predictive performance relative to NDVI-based linear models [68,69]."

**Page 22, Discussion section, line 472, now:**

"The KRR-based model for estimating LB severity (LB-KRR) further captured nonlinear relationships within multispectral reflectance data, consistent with the theoretical foundations of kernel-based methods for modeling complex, high-dimensional data [68,69]."

**Page 24, Discussion section, line 513, before:**

"The high coincidence observed for the top 10% of genotypes (SC10 = 65.4% in Trial A and 87.2% in Trial B with KRR) indicates that a limited number of acquisitions—and potentially even a single well-timed flight—can approximate AUDPC-based selections while reducing the need for repeated field assessments."

**Page 23, Discussion section, line 513, now:**

"The high coincidence observed for the top 10% of genotypes indicates that a limited number of acquisitions—and potentially even a single well-timed flight—can approximate AUDPC-based selections while reducing the need for repeated field assessments."

**Note:** The specific SC10 values (65.4% in Trial A and 87.2% in Trial B) were relocated from the Discussion to the Results section, where they are now reported alongside the other selection coincidence metrics, avoiding duplication between the two sections:

**Page 19, Results section, line 418, before:**

"In contrast, substantially higher SC values were observed in the second UAV assessment, ranging from 47.6% to 77.4% in Trial A and from 80.9% to 91.5% in Trial B (Fig 4)."

**Page 19, Result section, line 418, now**:

“In contrast, substantially higher SC values were observed in the second UAV assessment, ranging from 47.6% to 77.4% in Trial A and from 80.9% to 91.5% in Trial B, with SC10 values (i.e., selected coincidence within the top 10% of AUDPC-ranked genotypes) of 65.4% in Trial A and 87.2% in Trial B for the KRR-based estimates (Fig 4).”

**Reviewer #2:**

Image acquisition between 11:00 AM and 2:00 PM is justified by maximum non-photochemical quenching. However, the manuscript lacks details on how irradiance variations (e.g., passing clouds, which are common in Oxapampa) were handled during radiometric calibration, beyond the use of the reference panel.

Our response:

We thank the reviewer for this observation. In addition to the calibrated reference panel used for radiometric correction, irradiance stability during each flight session was monitored using a PAR (Photosynthetically Active Radiation) sensor connected to a LI-1400 datalogger (LI-COR, Lincoln, USA), which continuously recorded incident light throughout image acquisition.

For each of the four assessment dates (64 and 86 DAP in Trial A; 38 and 58 DAP in Trial B), multiple aerial image sets were acquired within the 11:00–14:00 h acquisition window. The PAR sensor readings were used to identify the image set with the most stable irradiance conditions (i.e., minimal variation caused by passing clouds), and this set was selected for radiometric correction and subsequent analysis. This procedure complemented the reference panel calibration by ensuring that the selected imagery corresponded to a period of consistent solar irradiance, reducing potential bias from transient cloud cover. We have clarified this procedure in the revised "Field late blight severity assessment, image acquisition, and preprocessing" subsection (see text below).

**Text added to manuscript:**

**Page 10, Materials and Methods section, line 213:**

“Given the frequent occurrence of passing clouds at the study site, irradiance stability during image acquisition was monitored in real time using a LI-190S quantum PAR sensor connected to a LI-1400 datalogger (LI-COR, Lincoln, USA). For each assessment date, multiple aerial image sets were acquired, and the set exhibiting the most stable irradiance conditions was selected for radiometric correction and subsequent analysis.”

The manuscript lacks details regarding pathogen inoculation, even if it is implied that the infection occurred naturally. A more detailed description of this procedure would be highly valuable. Is it possible for the authors to characterize the predominant clonal lineage or compatibility groups present in the experimental area?

Our response:

**Comment 1:** *The manuscript lacks details regarding pathogen inoculation, even if it is implied that the infection occurred naturally. A more detailed description of this procedure would be highly valuable.*

We thank the reviewer for this comment. No artificial inoculation with *Phytophthora infestans* was performed in either field trial. Disease development resulted exclusively from natural infection at the Oxapampa screening site (Pasco, Peru), a location traditionally used by the International Potato Center (CIP) to evaluate potato resistance to late blight due to its consistently high LB disease pressure (Pérez et al. 2001). We have expanded the "Study area" subsection of Materials and Methods to clarify that field trials were conducted on farmer’s field rented by CIP under a land-use agreement with the local landowner, given that Oxapampa has been traditionally used by CIP as a screening site for LB resistance (see text below).

As already described in the original "Study area" subsection, Oxapampa has a temperate, rainy, and very humid climate year-round, and during the trials, minimum and maximum temperatures ranged from 8.6 to 16.2 °C and 18.9 to 27.0 °C, respectively, with relative humidity between 69.0 and 94.7% and total growing-season precipitation of 559.6 mm (Trial A) and 894.7 mm (Trial B) (S1 Fig). We have further expanded this subsection to explicitly clarify that these conditions were sufficient to promote natural LB epidemics without the need for artificial inoculation (see text below).

In endemic screening locations with consistent natural epidemics, resistance may be evaluated using naturally occurring inoculum, provided that susceptible controls are included to verify disease establishment and epidemic progression (Forbes et al. 2014). In the present study, a common set of three varieties with known responses to LB — Amarilis (moderately resistant), Yungay (highly susceptible), and Kory (resistant) — was included in both trials as controls, as already described in the "Plant material and experimental design" subsection of Materials and Methods.

**Text added to manuscript:**

**Page 6, Materials and Methods section, line 129:**

"Field trials were conducted on farmer’s field rented by the International Potato Center (CIP) under a land-use agreement with the local landowner, as Oxapampa has been traditionally used by CIP as a screening site for LB resistance [5,15,26]."

**Page 7, Materials and Methods section, line 138:**

"These environmental conditions allowed evaluation of the potato materials under naturally occurring LB infection, without artificial inoculation.

**Page 7, Materials and Methods section, line 145:**

No governmental or special collection permits were required for this study."

**Comment 2:** *Is it possible for the authors to characterize the predominant clonal lineage or compatibility groups present in the experimental area?*

We thank the reviewer for this comment. The *Phytophthora infestans* population present in the experimental plots was not sampled or genotyped during either trial; therefore, its precise clonal composition cannot be directly confirmed.

However, previous studies characterizing *P. infestans* isolates from Oxapampa and other Peruvian potato-growing areas identified EC-1 (A1 mating type) as the predominant clonal lineage infecting cultivated potato [Pérez et al. 2001; Garry et al. 2005]. Subsequent nationwide surveillance confirmed that EC-1 remains dominant across Peru, exhibiting complex virulence and considerable subclonal variation [Lindqvist-Kreuze et al. 2020].

Based on this evidence, EC-1 (A1 mating type) was likely the predominant lineage at the experimental site during both trials, although this was not directly confirmed in the present study. We have clarified this as a limitation in the revised manuscript. This addition required citing two new references not previously included in our reference list — Garry et al. 2005 and Lindqvist-Kreuze et al. 2020 — which have been added to the References section accordingly.

**Text added to manuscript:**

**Page 6, Materials and Methods section, line 140:**

“The *Phytophthora infestans* population present during both trials was not sampled or genotyped; consequently, its precise clonal composition could not be determined. However, previous studies characterizing isolates from Oxapampa and other Peruvian potato-growing areas identified EC-1 as the predominant clonal lineage infecting cultivated potato, belonging to the A1 mating type and characterized by complex virulence and considerable subclonal variation [26,28,29].”

I suggests that the authors provide further details regarding the phenotypic evaluation performed by the raters. How were these raters trained? How were the scores assigned? Was a diagrammatic scale used?

Our response:

We thank the reviewer for this suggestion. Late blight severity was assessed by CIP personnel with extensive experience in phenotypic evaluation of potato resistance to Phytophthora infestans under field conditions, following the standardized CIP methodology described by Forbes et al. (2014).

As already stated in the manuscript, severity was visually estimated at the plot level as the percentage of LB-infected foliar area, recorded on a 0–100% scale in 5% increments. Evaluators estimated severity by visually comparing the proportion of healthy green foliage to the proportion affected by late blight. A categorical diagrammatic scale was not used; the CIP field assessment guide recommends direct percentage estimates over categorical or logarithmic scales, as the latter do not necessarily correct for evaluator bias, and recommends that assessments be performed by experienced evaluators to maintain consistency and precision across successive readings (Forbes et al. 2014).

We have expanded the "Field late blight severity assessment, image acquisition, and preprocessing" subsection to explicitly clarify rater expertise and the rationale for using a direct percentage scale. The following changes were made:

**Page 9, Materials and Methods section, line 187, before:**

“LB severity was assessed as the percentage of LB-infected foliar area per plant, visually estimated by trained experts assuming LB was the only foliar disease present in the plant, and recorded on a 0–100% scale in 5% increments [15].”

**Page 9, Materials and Methods section, line 187, now:**

“LB severity was assessed by CIP personnel with extensive experience in the field evaluation of potato resistance to late blight, following the standardized CIP methodology described by [15]. Severity was assessed as the percentage of LB-infected foliar area per plant, visually estimated by trained experts assuming LB was the only foliar disease present in the plant, and recorded on a 0–100% scale in 5% increments. A categorical diagrammatic scale was not used for these estimates, as direct percentage scoring is recommended by the CIP field assessment guide to minimize evaluator bias.”

I would like more clarity regarding why the visual evaluations were performed at a one-week interval. Shorter intervals would ensure greater accuracy in severity estimates. What led to the choice of a seven-day interval?

Our response:

Thank you for this observation. The seven-day interval was selected according to the standardized CIP methodology for field assessment of resistance to late blight (Forbes et al. 2014), which recommends adjusting evaluation frequency to the expected rate of epidemic development: every seven days in relatively cool locations such as Oxapampa, every three to four days in warmer, more humid environments with rapid disease progression, or every 10–14 days when development is slow. The goal is to capture observations spanning low, intermediate, and high disease levels across all genotypes, including susceptible controls.

The onset of field evaluations (41 DAP in Trial A and 38 DAP in Trial B) was determined by the appearance of initial late blight symptoms on the susceptible control (Yungay), which served as an indicator of natural epidemic onset. From that point, weekly assessments tracked disease progression consistently across all plots, yielding seven readings in Trial A and five in Trial B — sufficient to capture the principal phases of the epidemic and differentiate resistant from susceptible responses. This schedule was consistent with previous field trials conducted under natural late blight pressure at the Oxapampa screening site (Gastelo et al. 2022).

We agree that shorter intervals could offer additional temporal resolution, particularly in cases of very rapid epidemic development. However, strictly equal or very short intervals are not indispensable for AUDPC calculation, since the trapezoidal integration method already incorporates the actual time elapsed between observations. In this context, the seven-day interval represented an appropriate balance between epidemic monitoring, field logistics, and consistency across all experimental plots.

**Text added to the manuscript:**

**Page 9, Materials and Methods section, line 195:**

“The onset of assessments was determined by the appearance of initial late blight symptoms on the susceptible control (Yungay), and the weekly interval was selected according to the standardized CIP methodology for late blight field trials and the expected rate of epidemic development under the relatively cool conditions at the Oxapampa site [15].”
